# Adesão à Vacina Contra Influenza em Idosos com Comorbidades Cardiovasculares

**DOI:** 10.36660/abc.20240537

**Published:** 2025-03-27

**Authors:** Rodrigo S. Aguilar, Ana Paula Rosim Giraldes, Maria Paula Barbieri Delia, Meliza Goi Roscani, Henrique Pott

**Affiliations:** 1 Programa de Pós-Graduação em Biotecnologia Universidade Federal de São Carlos São Carlos SP Brasil Programa de Pós-Graduação em Biotecnologia - Universidade Federal de São Carlos (UFSCar), São Carlos, SP – Brasil; 2 Hospital Universitário Universidade Federal de São Carlos São Carlos SP Brasil Hospital Universitário da Universidade Federal de São Carlos (HU-UFSCar), São Carlos, SP – Brasil; 3 Programa de Pós-Graduação em Gerontologia Universidade Federal de São Carlos São Carlos SP Brasil Programa de Pós-Graduação em Gerontologia - Universidade Federal de São Carlos (UFSCar), São Carlos, SP – Brasil; 4 Departamento de Medicina Universidade Federal de São Carlos São Carlos SP Brasil Departamento de Medicina - Universidade Federal de São Carlos, São Carlos, SP – Brasil

**Keywords:** Influenza Humana, Vacinas, Idoso, Doenças Cardiovasculares.

## Abstract

**Fundamento:**

A vacina de influenza diminui a ocorrência de doenças e mortes em idosos, principalmente naqueles com comorbidades cardiovasculares.

**Objetivo:**

Investigar a adesão à vacina contra Influenza em pacientes brasileiros idosos que vivem na comunidade e possuem comorbidades cardiovasculares.

**Métodos:**

Este estudo transversal analisou dados da segunda onda do ELSI-Brasil (2019-2021), que compreendeu a participação de 9.949 idosos. Os participantes com doenças cardiovasculares relataram se haviam recebido a vacina contra Influenza no ano anterior. Foram identificados fatores relacionados à vacinação com análises de subgrupos para cada comorbidade cardiovascular. Realizou-se uma análise exploratória para identificar os principais fatores que levaram à não vacinação. A significância estatística foi definida como bicaudal p < 0,05.

**Resultados:**

Este estudo contou com a participação de 5.296 indivíduos. Dentre eles, 76,6% relataram ter recebido a vacina contra Influenza no ano anterior à coleta de dados. Os indivíduos vacinados eram, em geral, do sexo feminino, de idade mais avançada, viúvas, não fumantes, com hábitos de vida mais saudáveis e acesso a planos de saúde privados, apesar de apresentarem maior fragilidade e comorbidades cardiovasculares. Entre os subgrupos analisados, a idade teve um impacto significativo na probabilidade de vacinação. Entre os participantes hipertensos, o acesso a planos de saúde privados e uma boa condição de saúde aumentaram as chances de vacinação, enquanto o tabagismo e o consumo de álcool as reduziram. Os motivos mais frequentes para a recusa de vacinação contra Influenza foram o medo de reações adversas (18,2%), senso de baixo risco de infecção (14,9%), indisponibilidade da vacina (13,9%) e incerteza quanto à eficácia (12%).

**Conclusão:**

No Brasil, cerca de 24% de idosos com doenças cardiovasculares continuam não vacinados contra Influenza, representando sérios riscos à saúde. O fomento de estratégias que abordem crenças pessoais, ampliem o acesso à vacina e aumentem o engajamento dos profissionais de saúde é fundamental. As intervenções individualizadas devem considerar as características demográficas e de saúde da população a fim de superar esses obstáculos de forma eficaz.

## Graphical abstract

**Figura Central:**
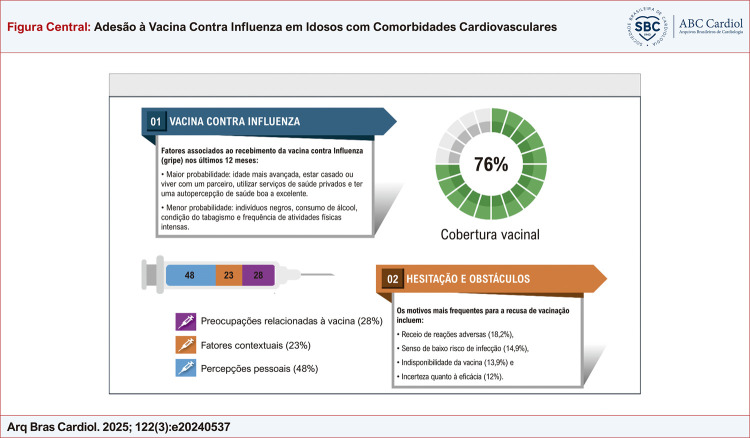
: Adesão à Vacina Contra Influenza em Idosos com Comorbidades Cardiovasculares

**Figura Central:** : Adesão à Vacina Contra Influenza em Idosos com Comorbidades Cardiovasculares

Adesão à vacina contra Influenza em idosos com comorbidades cardiovasculares.

## Introdução

A Influenza (gripe) é um vírus altamente contagioso e se espalha rapidamente por todo o mundo, afetando pessoas de qualquer idade.^1^ Entretanto, idosos, gestantes, crianças com menos de cinco anos e indivíduos com doenças crônicas estão mais suscetíveis a complicações e até mesmo ao óbito em decorrência do vírus.^2^ Segundo a Organização Mundial da Saúde (OMS), cerca de 5-15% dos adultos contraem Influenza anualmente, o que resulta em 3-5 milhões de casos graves e 250.000 a 500.000 mortes.^3^ Estudos recentes demostraram que a Influenza tem um impacto significativo nos serviços de saúde, representando um aumento notável nas consultas clínicas e hospitalares.^4 , 5^ Dados dos Estados Unidos destacam que uma grande parte das hospitalizações (54-70%) e óbitos (71-85%) relacionadas à gripe ocorrem em indivíduos com 65 anos ou mais.^3^ Além de representar riscos graves à saúde, a Influenza também gera uma sobrecarga financeira considerável, decorrente de gastos como consultas médicas, internações, medicamentos, entre outros custos relacionados.^4 - 6^

Diversos estudos demonstraram os efeitos positivos da vacina contra Influenza, mostrando benefícios significativos para a saúde e redução de custos.^7^ Esses achados são particularmente importantes para populações vulneráveis, como idosos, que apresentam um maior risco de doenças graves e complicações.^1^ Além disso, a incidência de doenças cardiovasculares aumenta com o avanço da idade, afetando tanto homens quanto mulheres.^8^ Segundo a literatura, a Influenza pode agravar consideravelmente as doenças cardiovasculares ao elevar a produção de citocinas pró-inflamatórias, podendo resultar na ruptura de placas ateroscleróticas e em eventos isquêmicos agudos posteriores.^9^ Entretanto, estudos recentes destacaram a importância da vacina contra Influenza na prevenção da piora clínica, diminuição da mortalidade geral e redução de mortes associadas a complicações cardiovasculares.^10^

Apesar desses riscos, muitos adultos em grupos de alto risco, especialmente aqueles com doenças cardiovasculares, optam por não se vacinar contra Influenza.^11 , 12^ Essas decisões são influenciadas por fatores como a autopercepção da condição de saúde, preocupações com os efeitos adversos da vacina, incertezas quando à eficácia e praticidade de receber a vacinação fora de hospitais ou clínicas.^11 - 13^Ademais, carecem dados sobre a cobertura vacinal entre idosos com doenças cardiovasculares e os fatores que influenciam suas escolhas em contextos comunitários.

No Brasil, o Ministério da Saúde disponibiliza a vacina contra Influenza de forma gratuita para idosos, com uma meta de cobertura vacinal de 90%.^14 , 15^ Contudo, essa meta não foi alcançada de forma consistente, o que indica áreas que precisam melhorar.^14^ A fim de entender melhor a adesão à vacina contra Influenza, utilizamos dados do estudo ELSI-Brasil para investigar os fatores determinantes que influenciam essa vacinação. O objetivo desse estudo é investigar a adesão à vacina contra Influenza em pacientes idosos brasileiros que vivem na comunidade e possuem comorbidades cardiovasculares, bem como explorar os motivos por trás da hesitação em se vacinar. Este estudo é de extrema importância, já que proporciona insights para otimizar as estratégias de saúde pública voltadas a idosos em grupos de alto risco, considerando os contextos de pesquisa atuais.

## Métodos

### Fonte de dados: Estudo Longitudinal da Saúde dos Idosos Brasileiros (ELSI-Brasil)

O ELSI-Brasil trata-se de uma pesquisa conduzida para analisar a dinâmica do envelhecimento da população brasileira e os fatores que a influenciam. Ele representa indivíduos com 50 anos ou mais de 70 municípios de todas as cinco regiões do Brasil.^16^ O estudo teve como objetivo determinar como os serviços sociais e de saúde podem beneficiar a população idosa. A primeira onda da pesquisa foi realizada entre 2015 e 2016 com 9.412 participantes, e a segunda onda se deu entre 2019 e 2021, contando com 9.949 participantes. A pesquisa foi aprovada pelo Comitê de Ética em Pesquisa do Instituto René Rachou, Fundação Oswaldo Cruz (CAAE: 34649814.3.0000.5091) e todos os participantes assinaram o termo de consentimento. Para mais informações e acesso aos dados, acesse a URL fornecida: http://elsi.cpqrr.fiocruz.br

### Desenho do estudo e participantes

**Figura 1:**
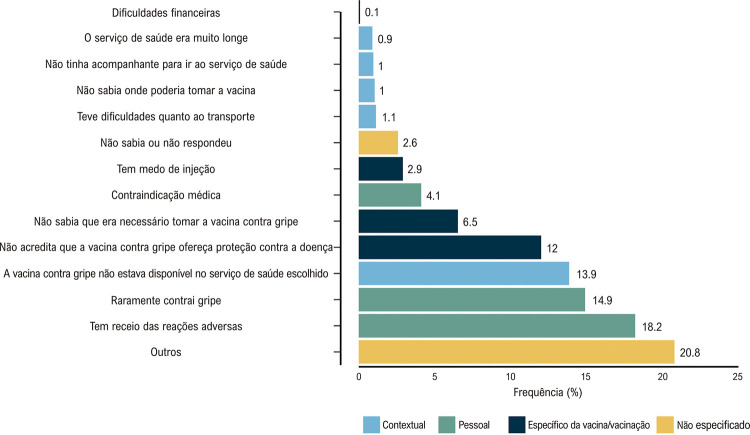
– Principais motivos para não administrar a vacina contra Influenza (N = 1.241).

Este estudo transversal utilizou os dados da segunda onda do ELSI-Brasil (2019-2021). O estudo incluiu indivíduos com 50 anos ou mais, diagnosticados com doenças cardiovasculares, que informaram se receberam a vacina contra Influenza no ano anterior. As doenças cardiovasculares consideradas para a análise foram hipertensão, pressão alta, angina estável crônica, infarto do miocárdio e insuficiência cardíaca. Os participantes responderam sobre seu histórico vacinal, em particular se haviam recebido a vacina contra Influenza no ano anterior. Foram excluídos os participantes com dados incompletos relativos a doenças cardiovasculares ou ao histórico vacinal contra Influenza no ano anterior. Indivíduos cujos dados ausentes ultrapassaram 20% das variáveis utilizadas para o desenvolvimento do índice de fragilidade também foram excluídos. O processo de seleção da população do estudo encontra-se ilustrado no Material Complementar ( Figura 1 ).

### Variáveis sociodemográficas e clínicas

As seguintes variáveis sociodemográficas e clínicas foram retiradas dos arquivos de dados públicos da segunda onda do ELSI-Brasil: idade, sexo, etnia/cor, estado civil, área de residência (urbana ou rural), status de tabagismo, consumo de álcool, participação em atividades físicas (tanto intensidade quanto frequência), autopercepção da saúde, doenças cardiovasculares específicas e o tipo de serviço de saúde (público ou privado).

### Comorbidades cardiovasculares

Utilizamos dados do questionário de autorrelato, coletados por meio de entrevistas individuais, para identificar indivíduos com doenças cardiovasculares. As perguntas específicas e os filtros aplicados para cada condição são os seguintes:

- *Hipertensão ou pressão alta:* Uma resposta positiva à pergunta: “n28 - Você já foi diagnosticado com hipertensão arterial (pressão alta) por algum médico?” Excluíram-se casos cujas respostas à pergunta sobre hipertensão ou pressão alta foram “Sim, apenas durante a gravidez”.
- *Angina estável crônica:* Uma resposta positiva à pergunta: “n48 - Você já foi diagnosticado com angina pectoris por algum médico?”
- *Infarto do miocárdio* : Uma resposta positiva à pergunta: “n46 - Você já foi diagnosticado com infarto do miocárdio (ataque cardíaco) por algum médico?”
- *Insuficiência cardíaca* : Uma resposta positiva à pergunta: “n50 - Você já foi diagnosticado com insuficiência cardíaca por algum médico?”

Todas essas condições foram agrupadas em uma classificação binária, “Comorbidades cardiovasculares” (sim/não). Como parte da análise exploratória, realizamos análises individuais para cada condição.

### Avaliação da cobertura vacinal contra influenza

Identificamos o status de vacinação contra Influenza dos participantes ao coletar as respostas à pergunta “n67 - Você recebeu a vacina contra gripe nos ÚLTIMOS 12 MESES?” Com base nas respostas afirmativas ou negativas, os participantes foram classificados, respectivamente, como “Vacinados” ou “Não vacinados”. Casos com dados ausentes foram excluídos das análises.

Para aqueles classificados como “Não vacinados”, também investigamos os principais motivos para a recusa da vacina contra a gripe, utilizando a pergunta “n68 - Qual foi o principal motivo para a recusa da vacina contra gripe?” As possíveis respostas para a pergunta eram: (1) Raramente contrai gripe; (2) Não sabia que era necessário tomar a vacina contra gripe; (3) Não sabia onde poderia tomar a vacina; (4) Tem receio das reações adversas; (5) Tem medo de injeção; (6) Não tinha acompanhante para ir ao serviço de saúde; (7) Enfrentava dificuldades financeiras; (8) Teve dificuldades quanto ao transporte; (9) O serviço de saúde era muito longe; (10) A vacina contra gripe não estava disponível no serviço de saúde escolhido; (11) Contraindicação médica; (12) Não acredita que a vacina contra gripe ofereça proteção contra a doença; (13) Outro motivo; (99) Não sabia ou não respondeu. Com o intuito de investigar os motivos por trás da hesitação vacinal, adotamos a definição do Grupo de Trabalho SAGE,^15^que descreve essa hesitação como o adiamento ou a recusa da vacina, mesmo quando os serviços estão disponíveis. As respostas foram divididas entre três tópicos principais: (1) fatores contextuais, (2) influências pessoais e (3) influências relacionadas especificamente à vacina contra Influenza.

### Avaliação de fragilidade

A fragilidade, que indica a vulnerabilidade dos participantes a desfechos negativos de saúde, foi avaliada por meio de um índice de fragilidade fundamentado na abordagem de déficits de saúde acumulados.^17^ Acreditamos que a fragilidade pode explicar o viés favorável à adesão à vacina da gripe,^18 , 19^ evidenciando a importância de ajustar nossos modelos de regressão para considerar o nível de fragilidade da população do estudo.

Recentemente, estabelecemos procedimentos padrão para a elaboração de um índice de fragilidade com base nos dados da segunda onda do ELSI-Brasil. Em suma, calculamos os escores de déficit de cada indivíduo com base no número total de déficits de saúde, incluindo doenças associadas à idade, incapacidades e funcionalidade. (Vide Material Complementar, Tabela 1 ). Esse escore foi, então, convertido em um Índice de Fragilidade (IF) dentro de uma escala de 0 a 1 (IF = escore de déficit/n, onde “n” é o número de componentes derivados de doenças relacionadas à idade, incapacidades e funcionalidade). Para fins práticos, o escore do IF foi categorizado entre três classes: não frágil (IF < 0,1), pré-frágil (IF > 0,1 e < 0,21) e frágil (IF > 0,21).^20^

**Tabela 1:** –Características sociodemográficas, clínicas e de vacinação da amostra do estudo

Variável	Nível	Total	Não vacinados	Vacinados	p
		N = 5.296	N = 1.241	N = 4.055	
**Idade, mediana [IIQ]**	Anos	67 [60, 75]	61 [56, 71]	68 [62, 76]	< 0,001
**Sexo, %**	Feminino	3.059 (57,8)	686 (55,3)	2.373 (58,5)	0,047
	Masculino	2.237 (42,2)	555 (44,7)	1.682 (41,5)	
**Etnia, %**	Branco	2.361 (44,6)	502 (40,5)	1.859 (45,8)	0.001
	Preto	619 (11,7)	174 (14,0)	445 (11,0)	
	Pardo	2.270 (42,9)	558 (45,0)	1.712 (42,2)	
	Amarelo/Indígena	28 (0,5)	3 (0,2)	25 (0,6)	
	Não responderam	18 (0,3)	4 (0,3)	14 (0,3)	
**Estado civil, %**	Solteiro	2.506 (47,3)	572 (46,1)	1.934 (47,7)	0,339
	Casado/Vive com o parceiro	2.790 (52,7)	669 (53,9)	2.121 (52,3)	
**Área de residência, %**	Rural	855 (16,1)	195 (15,7)	660 (16,3)	0,669
	Urbana	4.441 (83,9)	1.046 (84,3)	3.395 (83,7)	
**Consumo de álcool, %**	Nunca	4.318 (81,7)	969 (78,2)	3.349 (82,8)	< 0,001
	Menos de uma vez ao mês	407 (7,7)	93 (7,5)	314 (7,8)	
	Uma vez ao mês ou mais	559 (10,6)	177 (14,3)	382 (9,4)	
**Status de tabagismo, %**	Não tabagista	3.518 (66,4)	768 (61,9)	2.750 (67,8)	< 0,001
	Tabagista	564 (10,6)	188 (15,1)	376 (9,3)	
	Ex-tabagista	1.199 (22,6)	282 (22,7)	917 (22,6)	
	Não responderam	15 (0,3)	3 (0,2)	12 (0,3)	
**Pratica atividade física mais de uma vez por semana, %**	Intensidade baixa	2.284 (43,1)	529 (42,6)	1.755 (43,3)	< 0,001
	Intensidade moderada	991 (18,7)	244 (19,7)	747 (18,4)	
	Intensidade alta	459 (8,7)	144 (11,6)	315 (7,8)	
**Frequência de prática de atividades físicas intensas, como correr, nadar, andar de bicicleta, fazer aeróbica ou jogar tênis, %**	Mais de uma vez por semana	459 (8,7)	144 (11,6)	315 (7,8)	< 0,001
	Uma vez por semana	137 (2,6)	44 (3,5)	93 (2,3)	
	Uma a três vezes por mês	68 (1,3)	14 (1,1)	54 (1,3)	
	Raramente ou nunca	4.524 (85,4)	1.008 (81,2)	3.516 (86,7)	
	Não responderam	108 (2,0)	31 (2,5)	77 (1,9)	
**Serviço de saúde, %**	Público	4.262 (80,5)	1.052 (84,8)	3.210 (79,2)	< 0,001
	Privado	1.029 (19,4)	189 (15,2)	840 (20,7)	
	Não responderam	5 (0,1)	0 (0,0)	5 (0,1)	
**Status de autopercepção de saúde, %**	Muito ruim	304 (5,7)	76 (6,1)	228 (5,6)	0,036
	Ruim	816 (15,4)	202 (16,3)	614 (15,1)	
	Regular	2.225 (42,0)	530 (42,7)	1.695 (41,8)	
	Boa	1.645 (31,1)	350 (28,2)	1.295 (31,9)	
	Muito boa	222 (4,2)	53 (4,3)	169 (4,2)	
	Excelente	83 (1,6)	30 (2,4)	53 (1,3)	
	Não responderam	1 (0,0)	0 (0,0)	1 (0,0)	
**Comorbidades cardiovasculares,%**	Hipertensão	5.103 (96,4)	1.190 (95,9)	3.913 (96,5)	0,444
	Angina estável crônica	188 (3,5)	39 (3,1)	149 (3,7)	0,044
	Infarto do miocárdio	420 (7,9)	91 (7,3)	329 (8,1)	0,030
	Insuficiência cardíaca	425 (8,0)	95 (7,7)	330 (8,1)	0,416
**Avaliação de fragilidade**	Índice de fragilidade, mediana [IIQ]	0,20 [0,12, 0,32]	0,20 [0,12, 0,30]	0,21 [0,13, 0,32]	0,025
	Nível de fragilidade, %				
	Não-frágil	925 (17,5)	238 (19,2)	687 (16,9)	0,136
	Pré-frágil	1.737 (32,8)	410 (33,0)	1.327 (32,7)	
	Frágil	2.634 (49,7)	593 (47,8)	2.041 (50,3)	

### Análise estatística

O estudo comparou os respondentes “Vacinados” e “Não vacinados”. De acordo com o teste de normalidade de Shapiro-Wilk, as variáveis contínuas foram apresentadas como mediana (intervalo interquartil), e as variáveis categóricas, como frequência absoluta (frequência relativa). Inicialmente, foram realizadas análises não ajustadas, examinando de forma individual as variáveis sociodemográficas e clínicas. O teste do qui-quadrado foi utilizado para as variáveis categóricas, e o teste de Mann-Whitney, para variáveis contínuas.

Modelos de regressão logística multivariada foram aplicados para explorar os fatores associados à adesão à vacina contra Influenza nos 12 meses anteriores à coleta de dados. As variáveis que compõem o modelo foram selecionadas por meio do método stepwise, levando em consideração sua significância na análise univariada entre os grupos. Antes da modelagem, excluímos todos os casos de “Não respondido”, pois sua baixa frequência poderia comprometer a confiabilidade dos resultados. As análises de subgrupos foram conduzidas separadamente para cada comorbidade cardiovascular. Além disso, uma análise exploratória investigou os principais motivos para a recusa de vacinação contra gripe entre os indivíduos classificados como “Não vacinados”.

A significância estatística foi definida como bicaudal p < 0,05. Todas as análises foram realizadas no R, versão 4.4.0 (2024-04-24 ucrt) – “Puppy Cup”, utilizando o RStudio IDE (RStudio 2024.04.0+735 “Chocolate Cosmos” Release).

## Resultados

### Cobertura vacinal

Após a filtragem dos dados da segunda onda do ELSI-Brasil (N = 9.949) para excluir variáveis com mais de 20% de dados ausentes entre aquelas utilizadas na elaboração do índice de fragilidade (n = 48), aplicamos um segundo filtro para selecionar todos os participantes com 50 anos ou mais, diagnosticados com doenças cardiovasculares, que informaram se receberam a vacina contra Influenza no ano anterior (N = 5.296). Dentre esses, 4.055 (76,6%) relataram ter recebido a vacina contra Influenza no ano anterior à coleta de dados da segunda onda do ELSI-Brasil.

### Características demográficas

A Tabela 1 ilustra as diferenças significativas entre as coortes com base no status de vacinação contra a influenza. O grupo de idosos vacinados eram, em sua maioria, do sexo feminino, de idade mais avançada, viúvas, divorciadas ou separadas. Geralmente, essas pessoas tinham hábitos de vida mais saudáveis, como menor consumo de álcool e ausência de tabagismo. Por outro lado, idosos não vacinados demonstraram maior participação em atividades físicas intensas com uma frequência maior que uma vez por semana. Além disso, os idosos vacinados relataram receber atendimento de profissionais de saúde serviços privados e apresentaram uma autopercepção de saúde mais elevado. Apesar desses hábitos saudáveis, também apresentaram taxas mais altas de comorbidades cardiovasculares (exceto hipertensão) e um índice de fragilidade mais elevado.

### Fatores que influenciam a adesão à vacinação

A Tabela 2 apresenta os resultados da análise de regressão logística multivariada, categorizados de acordo com as comorbidades cardiovasculares. A adesão à vacina contra Influenza nos 12 meses anteriores à coleta de dados foi associada de forma significativa a diversos fatores. Observou-se uma maior probabilidade de adesão à vacina entre indivíduos de idade mais avançada, que estavam casados ou vivendo com um parceiro, fazendo uso de serviços de saúde privados e com uma autopercepção de saúde boa a excelente. Em contrapartida, fatores como ser da etnia negra, ter um consumo elevado de álcool, o status de tabagismo e a prática frequente de atividades físicas intensas foram associados a uma menor probabilidade de vacinação. Embora a análise de subgrupos por comorbidades cardiovasculares tenha mostrado resultados variados, a idade permaneceu significativamente associada a uma maior probabilidade de vacinação. Além da idade, vários fatores foram associados de forma significativa à adesão à vacinação em cada subgrupo. Em especial no subgrupo de hipertensos, o fato de depender de um serviço de saúde privado e ter uma autopercepção de saúde boa ou excelente esteve associado de forma positiva a maiores chances de vacinação. Por outro lado, fatores como ser da etnia negra, ter um histórico de consumo de álcool uma vez por mês ou mais, tabagismo ativo e a prática de atividades físicas intensas mais de uma vez por semana foram associados a uma menor probabilidade de vacinação. Essas associações não foram observadas nos subgrupos de angina estável crônica e infarto do miocárdio, enquanto apenas uma associação negativa com o tabagismo foi observada no subgrupo de insuficiência cardíaca.

**Tabela 2:** – Resultados da regressão logística multivariada estratificados por subgrupos específicos de comorbidades cardiovasculares

Variável	Total (N = 5.112)*	Comorbidades Cardiovasculares*			
		Hipertensão (N = 4.927)	Angina estável crônica^**1**^ (N = 179)	Infarto do miocárdio^**2**^ (N = 389)	Insuficiência cardíaca^**3**^ (N = 409)
**Idade, anos**	1,05 (1,05 - 1,06)	1,06 (1,05 - 1,06)	1,06 (1,01 – 1,11)	1,01 (0,99 – 1,04)	1,04 (1,01 – 1,07)
**Sexo**					
Feminino	Referência	Referência	Referência	Referência	Referência
Masculino	0,93 (0,80 – 1,08)	0,92 (0,80 – 1,07)	1,09 (0,45 – 2,66)	0,72 (0,41 – 1,27)	1,46 (0,83 – 2,56)
**Etnia**					
Branco	Referência	Referência	Referência	Referência	Referência
Preto	0,76 (0,61 – 0,93)	0,74 (0,60 – 0,92)	1,19 (0,31 – 4,57)	1,07 (0,49 – 2,35)	0,85 (0,38 – 1,92)
Pardo	0,92 (0,80 – 1,07)	0,91 (0,78 – 1,06)	1,58 (0,66 – 3,77)	0,89 (0,51 – 1,54)	1,07 (0,63 – 1,84)
Amarelo/Indígena	2,53 (0,75 – 8,51)	2,24 (0,66 – 7,60)	-	-	-
**Estado civil**					
Solteiro	Referência	Referência	Referência	Referência	Referência
Casado/Vive com o parceiro	1,17 (1,01 – 1,35)	1,16 (0,99 – 1,34)	1,34 (0,55 – 3,24)	1,44 (0,82 – 2,53)	1,38 (0,79 – 2,38)
**Área de residência**					
Rural	Referência	Referência	Referência	Referência	Referência
Urbana	0,93 (0,77 – 1,12)	0,94 (0,77 – 1,14)	3,45 (0,96 – 12,42)	0,36 (0,12 – 1,09)	0,90 (0,41 – 1,96)
**Consumo de álcool**					
Nunca	Referência	Referência	Referência	Referência	Referência
Menos de uma vez ao mês	1,15 (0,89 – 1,49)	1,13 (0,87 – 1,46)	0,23 (0,04 – 1,20)	1,62 (0,48 – 5,41)	0,62 (0,23 – 1,68)
Uma vez ao mês ou mais	0,78 (0,63 – 0,97)	0,79 (0,64 – 0,99)	0,57 (0,15 – 2,20)	0,78 (0,32 – 1,92)	0,77 (0,31 – 1,93)
**Status de tabagismo**					
Não tabagista	Referência	Referência	Referência	Referência	Referência
Tabagista	0,68 (0,55 – 0,83)	0,70 (0,57 – 0,87)	0,46 (0,13 – 1,58)	0,69 (0,34 – 1,40)	0,42 (0,20 – 0,88)
Ex-tabagista	0,91 (0,77 – 1,08)	0,90 (0,76 – 1,07)	1,00 (0,40 – 2,50)	0,94 (0,53 – 1,67)	1,34 (0,76 – 2,39)
**Frequência de prática de atividade física intensa**					
Raramente ou nunca	Referência	Referência	Referência	Referência	Referência
Uma a três vezes por mês	1,09 (0,59 – 2,02)	1,17 (0,62 – 2,20)	-	-	1,23 (0,13 – 11,40)
Uma vez por semana	0,72 (0,49 – 1,06)	0,71 (0,48 – 1,04)	-	0,94 (0,09 – 9,93)	1,39 (0,16 – 12,20)
Mais de uma vez por semana	0,72 (0,58 – 0,90)	0,74 (0,59 – 0,93)	0,82 (0,23 – 2,90)	0,34 (0,13 – 0,92)	0,68 (0,30 – 1,54)
**Serviço de saúde**					
Público	Referência	Referência	Referência	Referência	Referência
Privado	1,29 (1,08 – 1,56)	1,30 (1,08 – 1,58)	0,89 (0,34 – 2,31)	1,67 (0,85 – 3,30)	1,42 (0,75 – 2,71)
**Status de autopercepção de saúde**					
Muito ruim/Ruim	Referência	Referência	Referência	Referência	Referência
Regular	1,14 (0,94 – 1,39)	1,17 (0,95 – 1,42)	0,68 (0,26 – 1,77)	0,97 (0,53 – 1,77)	1,08 (0,59 – 1,96)
Boa/Excelente	1,28 (1,02 – 1,61)	1,33 (1,06 – 1,69)	0,48 (0,14 – 1,65)	1,52 (0,68 – 3,39)	1,10 (0,48 – 2,51)
Escore do índice de fragilidade	0,68 (0,37 – 1,25)	0,74 (0,40 – 1,37)	0,33 (0,01 – 9,15)	0,61 (0,10 – 3,92)	0,53 (0,08 – 3,47)

*As estimativas são apresentadas como Taxas de Probabilidades (Intervalos de Confiança de 95%). 1 Para evitar estimativas enviesadas resultantes de um número pequeno de observações, excluímos dois casos (“Etnia Amarela/Indígena” e “Uma a três vezes por mês” da seção sobre a frequência de prática de atividades físicas intensas) do subgrupo “Angina estável crônica”. 2 Para evitar estimativas enviesadas resultantes de um número pequeno de observações, excluímos dois casos (“Etnia Amarela/Indígena” e “Uma a três vezes por mês” da seção sobre a frequência de prática de atividades físicas intensas) do subgrupo “Infarto do miocárdio”. 3 Para evitar estimativas enviesadas resultantes de um número pequeno de observações, excluímos um caso (“Etnia Amarela/Indígena”) do subgrupo “Infarto do miocárdio”.

### Motivos para hesitação à vacinação

Idosos não vacinados apresentaram vários motivos principais para não terem recebido a vacina contra Influenza. Os sensos pessoais sobre a vacina representaram 37,2% dos motivos, enquanto influências contextuais, como barreiras geográficas, corresponderam a 17,9%. Motivos diretamente relacionados à vacina ou à vacinação foram responsáveis por 21,5% dos casos ( Figura Central ). Esses motivos incluíram receio quanto a reações adversas (18,2%), senso de baixo risco de infecção (14,9%), indisponibilidade da vacina (13,9%) e incerteza quanto à eficácia (12%). A Figura 1 apresenta um gráfico de barras que ilustra os principais motivos para a recusa de vacina contra Influenza entre os não vacinados.

## Discussão

Este estudo nacional proporciona insights valiosos sobre a cobertura vacinal contra Influenza em pacientes idosos brasileiros com comorbidades cardiovasculares. Apesar dos esforços do Ministério da Saúde para oferecer vacinas gratuitamente e uma meta de cobertura vacinal de 90%,^15^ 24% desse grupo de alto risco permanece não vacinado, o que representa grandes riscos à saúde. Observou-se uma maior probabilidade de adesão à vacina entre indivíduos de idade mais avançada, que estavam casados ou vivendo com um parceiro, fazendo uso de serviços saúde privados e com uma autopercepção de saúde boa a excelente. Em contrapartida, fatores como ser da etnia negra, ter um consumo elevado de álcool, tabagismo e a prática frequente de atividades físicas intensas foram associados a uma menor probabilidade de vacinação. As opiniões pessoais sobre as vacinas foram o principal fator influenciando as decisões quanto à vacinação, seguidas por fatores contextuais. Os motivos mais frequentes para a recusa de vacinação incluíram receio quanto a reações adversas, senso de baixo risco de infecção, indisponibilidade da vacina e incerteza quando à eficácia. Além da idade, certos fatores influenciaram de forma significativa a adesão à vacinação dentro dos subgrupos. No subgrupo de hipertensos, o uso de serviços de saúde privados e a percepção positiva de saúde aumentaram as chances de vacinação. Por outro lado, ser da etnia negra, consumir álcool pelo menos uma vez ao mês ou mais, tabagismo e a prática frequente de atividades físicas intensas estavam associados a menores chances de vacinação. Esses padrões não foram observados nos grupos de angina estável crônica e infarto do miocárdio; apenas o tabagismo afetou negativamente a adesão à vacinação no subgrupo de insuficiência cardíaca.

Nossa análise revelou que os indivíduos vacinados eram, em sua maioria, do sexo feminino, de idade mais avançada, que fazem uso de serviços de saúde privados e têm uma autopercepção positiva de saúde. Esse padrão destaca a influência crítica do acesso à saúde e das percepções individuais de saúde nos comportamentos de vacinação.^21^ Esses achados concordam com a literatura existente, que enfatiza como os adultos, especialmente aqueles que utilizam serviços de saúde privados, têm mais propensão a perceber os benefícios da vacinação e agir de acordo.^22^ Os idosos conseguem reconhecer o aumento do risco de complicações pela Influenza e, assim, priorizar a vacinação. Em contrapartida, o acesso ao serviço de saúde privado pode proporcionar interações mais regulares com profissionais de saúde, que podem recomendar a vacinação.^22 , 23^ Além disso, uma autopercepção positiva de saúde pode estar correlacionada com comportamentos proativos em relação à saúde, o que pode aumentar a adesão à vacinação. Dados semelhantes foram observados em indivíduos com 60 anos ou mais (n = 1.224) que participaram do Estudo SABE 2015 (Saúde, Bem-Estar e Envelhecimento).^24^

Por outro lado, encontramos uma cobertura vacinal mais baixa em demografias que incluíam indivíduos negros, naqueles com maior consumo de álcool, tabagistas e que praticam atividades físicas intensas. A baixa cobertura vacinal entre indivíduos negros pode indicar desigualdades sociais e uma maior vulnerabilidade, ressaltando a necessidade de apoio direcionado para atender de forma eficaz às necessidades específicas.^22^ Isso destaca a importância de iniciativas de saúde pública culturalmente sensíveis e inclusivas, que possam alcançar e apoiar efetivamente essa demografia e garantir acesso igualitário a recursos de saúde e serviços de vacinação. De forma semelhante, a relação entre o alto consumo de álcool, o tabagismo e a hesitação em se vacinar sugere que intervenções direcionadas são necessárias para promover a vacinação nesses grupos.^25 , 26^ Curiosamente, aqueles que praticam atividades físicas intensas podem acreditar que são menos suscetíveis a doenças devido à boa autopercepção de saúde, o que exige um maior enfoque na educação dessas populações sobre a importância da vacinação, mesmo com seus estilos de vida saudáveis.^27^

Para indivíduos hipertensos, o acesso ao serviço de saúde privado e a autopercepção positiva de saúde estiveram correlacionados com uma maior probabilidade de adesão à vacina contra Influenza. Isso sugere que a condição de saúde percebida e a acessibilidade aos recursos de saúde são fundamentais para promover a vacinação dentro desse grupo. Por outro lado, taxas mais baixas de vacinação foram associadas a ser da etnia negra, ter um consumo frequente de álcool, tabagismo e prática de atividades físicas intensas. Esses achados podem indicar influências socioeconômicas e culturais veladas, incluindo possíveis obstáculos ao acesso à saúde ou diferenças nas crenças sobre saúde que desencorajam a vacinação. Curiosamente, essas associações não foram observadas nos subgrupos de angina estável crônica e infarto do miocárdio, sugerindo que a interação desses fatores pode variar entre as diferentes doenças cardiovasculares. No subgrupo de insuficiência cardíaca, apenas o tabagismo mostrou uma associação negativa, destacando o tabagismo como um determinante de importância no comportamento quanto à saúde nesse grupo.

Os achados deste estudo alinham-se com as políticas globais de saúde: Em 2003, a Assembleia Mundial da Saúde (resolução WHA 56.19) recomendou aumentar a cobertura vacinal contra Influenza para indivíduos no grupo de alto risco, com a meta de atingir uma cobertura de ≥ 75% entre os idosos e aqueles com doenças crônicas até 2010.^28^ No Brasil, o Ministério da Saúde disponibiliza a vacina contra Influenza de forma gratuita para idosos, com uma meta de cobertura vacinal de 90% até 2024.^14^ Segundo nossos achados, o Brasil alcançou com sucesso a meta de vacinar 75% dos idosos com comorbidades cardiovasculares. Dados semelhantes foram observados em indivíduos com 60 anos ou mais (n = 23.815) que participaram da Pesquisa Nacional de Saúde (PNS)^29^ de 2013 e do Estudo SABE de 2015.^24^ No entanto, são necessárias mais informações para determinar se isso foi alcançado em 2010 ou se nossos achados refletem uma conquista recente. Em especial, a adesão à vacina contra a influenza superou o limiar de 75% para todas as comorbidades cardiovasculares específicas estudadas, o que foi mais alto do que a adesão de 50-60% entre os idosos do grupo de alto risco em outros países.^12 , 30 - 32^ Apesar desses esforços, a meta de 90% não foi alcançada de forma consistente, o que indica áreas que precisam melhorar. Essas informações sugerem que, embora o Brasil tenha feito progressos significativos na cobertura vacinal para indivíduos no grupo de alto risco, ainda existe o potencial para melhorias adicionais a fim de atingir as metas nacionais.

Além disso, alcançar as metas de vacinação depende de incentivar a adesão e entender os motivos que impulsionam a hesitação vacinal entre os indivíduos não vacinados. A hesitação vacinal continua sendo uma questão complexa, influenciada por diversos fatores velados, relacionados à confiança e aos riscos percebidos.^33^ Um estudo inicial sobre a hesitação em relação à vacina contra a pólio, realizado por Rosenstock et al.,^34^ identificou fatores que continuam sendo relevantes. Esses fatores incluem a percepção da probabilidade da doença, o julgamento da gravidade da doença, a avaliação da eficácia da vacinação e as preocupações e influências que afetam a tomada de decisão. Nossos resultados mostraram características semelhantes entre os idosos com comorbidades cardiovasculares: receio quanto a reações adversas, senso de baixo risco de infecção e incerteza quanto à eficácia da vacina, destacando uma comunicação inadequada sobre a Influenza e a vacinação.

Além disso, um achado significativo foi que 13,9% dos participantes citaram a indisponibilidade da vacina como um obstáculo, destacando os desafios logísticos para alcançar uma cobertura vacinal ideal. Superar esses obstáculos exige planejamento estratégico, distribuição eficiente e uma coordenação otimizada entre os profissionais de saúde e as agências governamentais, a fim de garantir a disponibilidade constante, em especial durante períodos de alta demanda.^35 - 37^

Vários estudos demonstraram que os profissionais de saúde são de grande importância na abordagem e redução da hesitação vacinal por meio de uma comunicação clara e eficaz.^38 - 40^ Profissionais de saúde bem informados e treinados sobre vacinas estão mais propensos a recomendar a vacinação.^23 , 40^ No entanto, pode ser necessário um apoio adicional para lidar com conversas desafiadoras com pacientes ou familiares relutantes em se vacinar, onde o aval social e o apoio de colegas se tornam essenciais.^23^ A figura central apresenta, de forma resumida, como os profissionais de saúde podem abordar a hesitação vacinal.

Para lidar com essas questões, é importante investigar como os idosos, especialmente aqueles propensos a permanecer não vacinados, recebem informações sobre Influenza e a vacinação. Nosso estudo forneceu alguns insights sobre a população não vacinada, mas destacou a necessidade de informações mais detalhadas sobre como os idosos e suas famílias são informados. Estudos posteriores devem investigar a adesão à vacina contra Influenza em subgrupos específicos de idosos e pessoas com doenças crônicas.

Apesar dos insights fornecidos, nosso estudo possui várias limitações. O desenho transversal limita nossa capacidade de estabelecer relações causais entre o status de vacinação e as variáveis exploradas. O viés de memória pode afetar a precisão dos dados de vacinação autorrelatados, potencialmente impactando as taxas relatas. Além disso, a dependência de dados autorrelatados sobre doenças cardiovasculares pode representar viés de relato. Os critérios de exclusão, focados nos brasileiros com doenças cardiovasculares e dados ausentes, podem limitar a generalização de nossos achados para uma população mais ampla. No entanto, o uso de uma amostra nacional aumenta a confiabilidade e a aplicabilidade de nossos resultados, informando, assim, políticas e intervenções de saúde pública. Ademais, é importante considerar que a coleta de dados de 2019 a 2021 coincidiu com a pandemia de COVID-19, o que provavelmente influenciou os achados. A pandemia afetou o acesso aos serviços de saúde, os comportamentos de busca por atendimento e as prioridades de vacinação, impactando a adesão à vacina contra Influenza.^41 , 42^

## Conclusões

Em conclusão, nosso estudo revelou que uma parcela significativa de idosos com comorbidades cardiovasculares no Brasil permaneceu não vacinada contra Influenza. Isso ressalta a necessidade de intensificar os esforços de vacinação nessa população vulnerável. Nossos achados sugerem que estratégias personalizadas, que abordem crenças pessoais, dificuldades quanto ao acesso e o envolvimento dos profissionais de saúde, são essenciais para melhorar a vacinação contra a influenza. Ao alinhar de forma mais precisa as iniciativas dos profissionais de saúde com as características demográficas e de saúde específicas dos indivíduos, é possível mitigar os obstáculos prevalentes à vacinação observados neste estudo.

## *Material suplementar

Para informação adicional, por favor, clique aqui


