# Supplementary material for: Adesão à Vacina Contra Influenza em Idosos com Comorbidades Cardiovasculares
Source: Arq Bras Cardiol. 2025 Mar 27;122(3):e20240537. [Article in Portuguese] doi: 10.36660/abc.20240537 (PMC12061342; doi:10.36660/abc.20240537)
Supplement: Supplementary file 1 [file 0066-782X-abc-122-3-e20240537-suppl01.pdf]

Supplementary Material

This document contains supplementary material for the study "***Uptake of influenza vaccine among older adults with cardiovascular comorbidities***." The following pages include additional resources and information related to the article:

Contents

Figure 1. The study population selection process. 2
Table 1. Items included in the FI. 3

Figure 1. The study population selection process.

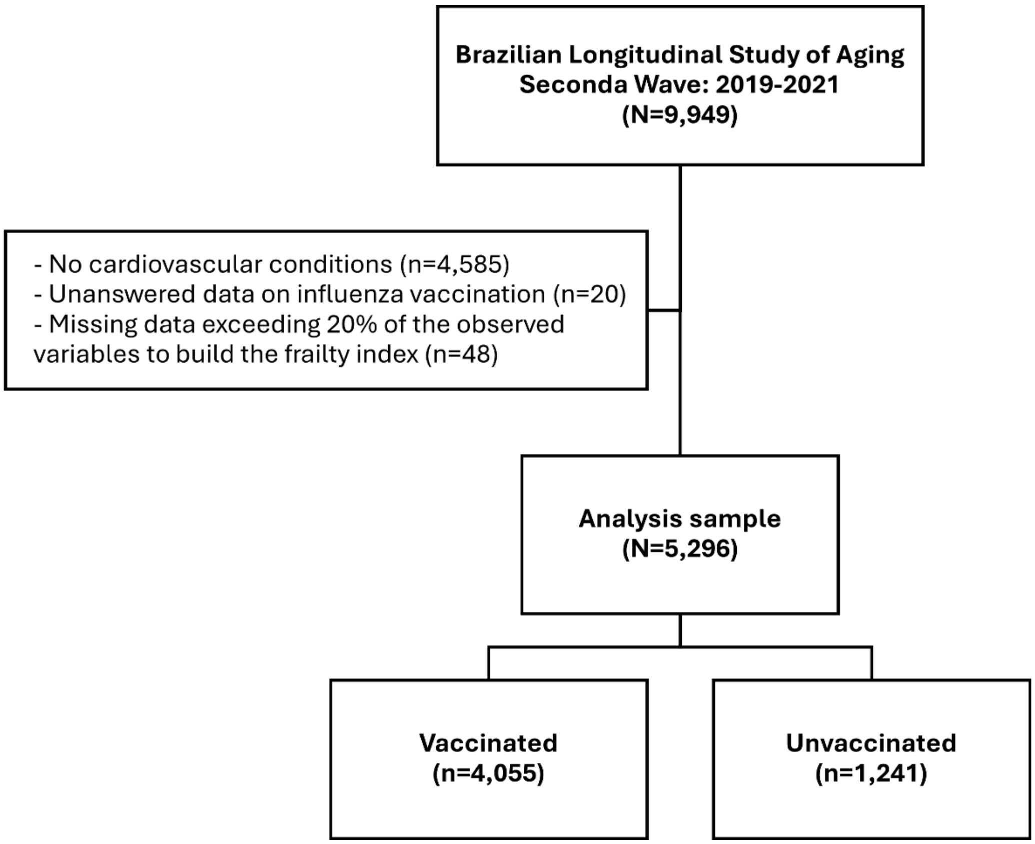

Table 1. Items included in the FI.

#	Item	Description
	General health and diseases	
1	n1	In general, how would you evaluate your health?
2	n6	How do you evaluate your far vision (EVEN WHEN USING GLASSES OR CONTACT LENSES), that is, recognizing someone that you know on the other side of the street at a distance of 65 feet or so?
3	n7	How do you evaluate your near vision (EVEN WHEN USING GLASSES OR CONTACT LENSES), this means recognizing an object that is within reach or reading a newspaper?
4	n16	How do you evaluate your hearing (EVEN WHEN USING A HEARING DEVICE)?
5	n28	Has any doctor ever told you that you have arterial hypertension (high blood pressure)?
6	n35	Has any doctor ever told you that you have diabetes ("high blood sugar")?
7	n44	Has any doctor ever told you that you have high cholesterol?
8	n46	Has any doctor ever told you that you had a heart attack?
9	n48	Has any doctor ever told you that you have angina pectoris?
10	n50	Has any doctor ever told you that you have a heart failure?
11	n52	Has a doctor ever told you that you had a cerebral vascular accident (stroke)?
12	n56	Has a doctor ever told you that you have arthritis or rheumatism?
13	n57	Has a doctor ever told you that you have osteoporosis?
14	n60	Has a doctor ever told you that you have or had cancer?

15	n62	Has a doctor ever told you that you have Parkinson's disease?
16	n63	Has a doctor ever told you that you have Alzheimer's disease?
17	n69	In the PAST THREE MONTHS, have you lost weight without any dieting?
18	n72	In the LAST WEEK, how often have you felt you could not handle your activities (started something, but could not finish it)?
19	n73	In the PAST WEEK, how often have your daily activities required a big effort from you?
20	n74	How would you evaluate the quality of your sleep?
21	n74_1	How often do you have trouble falling asleep (laying down and sleeping)?
22	n74_2	How often do you have trouble sleeping because you wake up during the night?
23	n74_3	How often do you have trouble sleeping because you wake up too early and can't go back to sleep?
	Mobility	
24	p5	Do you have difficulty running or jogging one kilometer or 10 blocks?
25	p6	Do you have difficulty walking one kilometer continuously?
26	p7	Do you have difficulty walking 100 meters (one block)?
27	p8	Do you have difficulty climbing SEVERAL flights of stairs WITHOUT RESTING?
28	p9	Do you have difficulty climbing ONE flight of stairs WITHOUT STOPPING or resting?
29	p10	Do you have difficulty sitting still for about two hours?

30	p10_1	Do you have difficulty getting up from a chair after sitting for a long time?
31	p12	Do you have difficulty bending over, kneeling or crouching?
32	p13	Do you have difficulty with extending one or both arms above shoulder level?
33	p14	Do you have difficulty with pulling or pushing large objects, such as an armchair?
34	p15	Do you have difficulty with lifting or carrying weights heavier than 5 kilograms, like a heavy bag of groceries?
35	p16	Do you have difficulty picking up a coin from a table? (Can't drag the coin to pick it up)
	Instrumental Activities of Daily Living	
36	p20	Do you have any difficulty with preparing A HOT MEAL?
37	p24	Do you have any difficulty with USING ANY TYPE OF TRANSPORTATION as a passenger?
38	p26	Do you have any difficulty with DOING SHOPPING?
39	p28	Do you have any difficulty with USING TELEPHONE (LANDLINE OR CELLULAR)?
40	p30	Do you have any difficulty with TAKING/MANAGING YOUR OWN MEDICATIONS?
41	p31_1	Do you have any difficulty with WALKING AROUND YOUR HOUSE OR IN THE GARDEN?
42	p22	Do you have any difficulty with MANAGING YOUR OWN MONEY?
43	p33	Do you have any difficulty with PERFORMING LIGHT HOUSEKEEPING (making your own bed, removing dust, taking care of the garbage etc.)?
44	p35	Do you have any difficulty with PERFORMING HEAVY HOUSEKEEPING?

	Basic Activities of Daily Living	
45	p37	Do you have any difficulty with GETTING ACROSS A ROOM OR WALKING FROM ONE ROOM TO ANOTHER on the same floor?
46	p40	Do you have any difficulty with DRESSING UP?
47	p17	Do you have any difficulty with DOING YOUR OWN PERSONAL HYGIENE?
48	p43	Do you have any difficulty with SHOWERING?
49	p46	Do you have any difficulty with EATING from a dish that was placed in front of you?
50	p49	Do you have any difficulty with GETTING IN OR OUT OF BED?
51	p55	Do you have any difficulty with USING THE BATHROOM?
52	p58	In the LAST MONTH, have you ever lost control of urine, unintentionally?
53	p58	In the LAST MONTH, have you ever lost control of feces, unintentionally?
