# Supplementary material for: Adesão à Vacina Contra Influenza em Idosos com Comorbidades Cardiovasculares
Source: Arq Bras Cardiol. 2025 Mar 27;122(3):e20240537. [Article in Portuguese] doi: 10.36660/abc.20240537 (PMC12061342; doi:10.36660/abc.20240537)
Supplement: Translation

# Translation: Uptake of Influenza Vaccine among Older Adults with Cardiovascular Comorbidities

**DOI:** 10.36660/abc.20240537i

**Authors:** Rodrigo S. Aguilar, Ana Paula Rosim Giraldes, Maria Paula Barbieri Delia, Meliza Goi Roscani, Henrique Pott

**Affiliations:** 1 Brazil Programa de Pós-Graduação em Biotecnologia - Universidade Federal de São Carlos (UFSCar), São Carlos, SP – Brazil; 2 Brazil Hospital Universitário da Universidade Federal de São Carlos (HU-UFSCar), São Carlos, SP – Brazil; 3 Brazil Programa de Pós-Graduação em Gerontologia - Universidade Federal de São Carlos (UFSCar), São Carlos, SP – Brazil; 4 Brazil Departamento de Medicina - Universidade Federal de São Carlos, São Carlos, SP – Brazil

**Keywords:** Human Influenza, Vaccines, Aged, Cardiovascular Diseases

## Abstract

**Background:**

Influenza vaccination reduces illness and fatality in older adults, especially those with cardiovascular comorbidities.

**Objective:**

To investigate influenza vaccination uptake among community-dwelling older Brazilian patients with cardiovascular comorbidities.

**Methods:**

This cross-sectional study analyzed data from the ELSI-Brazil Second wave (2019-2021), involving 9,949 older adults. Participants with cardiovascular conditions provided data on influenza vaccination from the previous year. Vaccination-associated factors were identified, with subgroup analyses for each cardiovascular comorbidity. An exploratory analysis investigated the primary reasons for non-vaccination. Statistical significance was determined with a two-sided P-value < 0.05.

**Results:**

This study included 5,296 individuals. Of these, 76.6% reported receiving the influenza vaccine within the year before data collection. Vaccinated individuals were generally older females, widowed, and non-smokers with healthier habits and private healthcare access, although they had higher frailty and cardiovascular comorbidities. Age significantly influenced the likelihood of vaccination across subgroups. In hypertension, private healthcare and good health status increased the odds, while smoking and alcohol consumption reduced them. The most common reasons for not receiving the influenza vaccine were fear of adverse reactions (18.2%), belief in a low-risk infection (14.9%), vaccine unavailability (13.9%), and lack of confidence in its effectiveness (12%).

**Conclusion:**

About 24% of older Brazilian adults with cardiovascular conditions remain unvaccinated against influenza, posing severe health risks. Strategies addressing personal beliefs, improving access, and enhancing healthcare provider engagement are crucial. Tailored interventions should align with the population’s demographic and health characteristics to overcome these barriers effectively.

## Introduction

Influenza is a highly contagious virus that spreads rapidly worldwide and affects people of all ages.^1^However, older adults, pregnant women, children under five years of age, and those with chronic conditions are more vulnerable to severe complications and even death from the virus.^2^The World Health Organization (WHO) reports that approximately 5-15% of adults catch influenza each year, resulting in 3-5 million severe cases and 250,000 to 500,000 fatalities.^3^Recent studies have shown that influenza significantly affects health services, with a notable increase in clinic and hospital visits due to illness.^4 , 5^Data from the United States highlight that a large proportion of flu-related hospitalizations (54–70%) and deaths (71–85%) occur in individuals aged ≥ 65 years.^3^to severe health risks, influenza also imposes a considerable financial burden associated with expenses, such as doctor appointments, hospital stays, medications, and other related costs.^4 - 6^

Numerous studies have demonstrated the positive effects of influenza vaccination, revealing significant health benefits and reduced economic costs.^7^These findings are particularly crucial for vulnerable populations, such as older adults, who are at increased risk of severe illness and related complications.^1^Furthermore, the incidence of cardiovascular disease increases with age and affects both men and women.^8^The literature indicates that influenza can significantly exacerbate cardiovascular conditions by increasing the production of pro-inflammatory cytokines, which can lead to the rupture of atherosclerotic plaques and subsequent acute ischemic events.^9^Nonetheless, recent studies have underscored the vital role of influenza vaccination in reducing clinical deterioration, decreasing overall mortality, and lowering deaths related to cardiovascular complications.^10^

Despite these risks, many high-risk adults, especially those with cardiovascular disease, skip vaccination against influenza.^11 , 12^Their decisions are influenced by factors, such as how they perceive their health status, concerns about vaccine side effects, doubts about vaccine efficacy, and considerations of convenience when receiving vaccinations outside hospitals or clinics.^11 - 13^Additionally, there is a lack of information on vaccination rates among older adults with cardiovascular diseases and the factors that drive their choice in community settings.

In Brazil, the Ministério da Saúde provides an influenza vaccine free of charge to older adults, with a target coverage rate of 90%.^14 , 15^However, this target has not been consistently achieved, indicating areas for improvement.^14^To better understand influenza vaccination uptake, we used data from the ELSI-Brazil study to investigate the determinants influencing this vaccination. We aimed to investigate influenza vaccination uptake among community-dwelling older Brazilian patients with cardiovascular comorbidities and explore the reasons for vaccine hesitancy. This study is crucial, as it offers insights into optimizing public health strategies for high-risk older adults within existing research contexts.

## Methods

Data source: The Longitudinal Study of Health in the Brazilian Elderly (ELSI-Brazil)

The ELSI-Brazil is a survey conducted to analyze the aging dynamics of the Brazilian population and the factors that influence it. It represents individuals aged 50 years or older residing in 70 municipalities across all five Brazilian regions.^16^The study aimed to determine how social and health services can benefit the aging population. The initial survey was conducted in 2015-2016 with 9,412 participants, and the second wave occurred between 2019-2021 with 9,949 participants. The survey was approved by the Ethics Review Committee of the Instituto René Rachou, Fundação Oswaldo Cruz (CAAE: 34649814.3.0000.5091), and all participants provided consent. For more information and access to the data, visit the provided URL: http://elsi.cpqrr.fiocruz.br

### Study design and participants

This cross-sectional study used data from the ELSI-Brazil Second wave (2019-2021). The study included people aged ≥ 50 years who were diagnosed with cardiovascular conditions and had information available about their influenza immunization in the past year. The cardiovascular conditions considered for the analysis were hypertension, high blood pressure, chronic stable angina, myocardial infarction, and heart failure. Participants were asked about their immunization history, specifically whether they had received an influenza vaccine in the past year. We did not include participants with incomplete data on their cardiovascular conditions or history of influenza immunization in the previous year. We also excluded individuals with significant missing data exceeding 20% of the variables used to build the frailty index. The study population selection process is illustrated in Supplementary Material ( Figure 1 ).

**Figure 1:** – Main reasons for not administering the influenza vaccine (N = 1,241).

### Sociodemographic and clinical variables

The following sociodemographic and clinical variables were extracted from the publicly available data files of the ELSI-Brazil second wave: age, sex, race, marital status, area of residence (urban or rural), smoking status, alcohol consumption, engagement in physical activities (both intensity and frequency), self-assessed health status, specific cardiovascular conditions, and type of health care provider (public versus private).

### Cardiovascular comorbid conditions

We used self-report questionnaire data from individual interviews to identify individuals with cardiovascular-related conditions. The specific questions and applied filters for each condition are as follows:

- *Hypertension or high blood pressure* : A positive response to the question: “n28 - Has any doctor ever told you that you have arterial hypertension (high blood pressure)?”. We excluded cases that responded “Yes, only during pregnancy” to the hypertension or high blood pressure question.
- *Chronic stable angina* : A positive response to “n48 - Has any doctor ever told you that you have angina pectoris?”.
- *Myocardial infarction* : A positive response to “n46 - Has any doctor ever told you that you had a heart attack?”.
- *Heart failure* : A positive response to “n50 - Has any doctor ever told you that you have heart failure?”.

We categorized all these conditions into a binary classification, “Cardiovascular Comorbid Conditions” (yes/no). We conducted separate analyses for each condition as part of an exploratory analysis.

### Influenza vaccination status assessment

We identified participants’ influenza vaccination status by extracting data from the question “n67 - In the LAST 12 MONTHS, have you taken the flu vaccine?”. Participants were categorized as “Vaccinated” if they answered positively and “Unvaccinated” if they responded negatively. Cases with missing data were excluded from the analyses.

For those classified as “Unvaccinated,” we also examined the main reasons for not taking the flu vaccine using the question “n68 - What was the main reason for not taking the flu vaccine?”. The available responses to the question were: (1) Rarely gets the flu; (2) Didn’t know it was necessary to take the flu vaccine; (3) Didn’t know where to take the vaccine; (4) Fear of vaccine reaction; (5) Fear of injection; (6) Didn’t have anyone to go with him/her to the health service; (7) Had financial difficulties; (8) Had transportation difficulties; (9) The health service was very far; (10) The flu vaccine was not available at the health service that he/she went to; (11) Medical contraindication; (12) Doesn’t believe that the flu vaccine protects against the flu; (13) Other; (99) Didn’t know/didn’t answer. To investigate the reasons behind vaccine hesitancy, we adopted the definition provided by the SAGE Working Group,^15^which describes vaccine hesitancy as the delay in accepting or refusing vaccines despite the availability of vaccination services. The responses were divided into three main topics: (1) contextual factors, (2) individual influences, and (3) influences specific to vaccines/vaccinations for influenza.

### Frailty assessment

Frailty, which indicates the participants’ vulnerability to adverse health outcomes, was quantified using a frailty index based on the accumulated health deficit approach.^17^We believe that frailty could explain the bias in favor of receiving the flu vaccine^18 , 19^highlighting the necessity of adjusting our regression models to accommodate the frailty level of the study population.

We recently outlined standard procedures for developing a frailty index using data from the ELSI-Brazil second wave. In summary, we used the total number of health deficits, including age-related diseases, disabilities, and functionality, to compute an individual’s deficit scores. (see Supplemental Material , Table 1 ) This score was then converted into a Frailty Index (FI) on a scale from 0 to 1 (FI = deficit score/n, where n is the number of components derived from age-related diseases, disabilities, and functionality). For practical purposes, we categorized the FI score into three classes: non-frail (FI < 0.1), pre-frail (FI > 0.1 and < 0.21), and frail (FI > 0.21).^20^

### Statistical analysis

The study compared “Vaccinated” and “Unvaccinated” respondents. According to the Shapiro-Wilk normality test, continuous variables were presented as median [interquartile range], and categorical variables were presented as absolute frequency (relative frequency). Initially, unadjusted analyses were conducted by individually examining the sociodemographic and clinical variables. The chi-square test was used for categorical variables, and the Mann–Whitney U test was used for continuous variables.

Multivariate logistic regression models explored factors associated with receiving an influenza vaccine 12 months before data collection. The variables included in the model were selected using a stepwise approach based on their significance in the univariate analysis between the groups. We excluded all cases with ‘Unanswered’ responses before modeling owing to their low count, which could potentially affect the results. Subgroup analyses were conducted separately for each comorbid cardiovascular condition. Additionally, an exploratory analysis investigated the primary reasons for not receiving the flu vaccine among those classified as “Unvaccinated.”

Statistical significance was assessed using a two-sided P-value < 0.05. All analyses were performed in R version 4.4.0 (2024-04-24 ucrt) -- “Puppy Cup” using RStudio IDE (RStudio 2024.04.0+735 “Chocolate Cosmos” Release).

## Results

### Vaccination rates

After filtering the second-wave data from ELSI-Brazil (N = 9,949) to exclude variables with missing data in more than 20% of the variables used to build the frailty index (n = 48), we applied a second filter to select all participants aged ≥ 50 years who were diagnosed with cardiovascular conditions and had information available about their influenza immunization in the past year (N = 5,296). Of these, 4,055 (76.6%) reported receiving influenza vaccination within the year prior to the ELSI-Brazil second-wave data collection.

### Demographic characteristics

Table 1 illustrates the significant differences between the cohorts based on their influenza immunization status. Vaccinated older adults were typically older females who were widowed, divorced, or separated. They generally exhibited healthier habits such as decreased alcohol consumption and non-smoking habits. Conversely, older adults who were not vaccinated showed higher participation rates in vigorous physical activity more than once per week. Additionally, vaccinated older adults reported receiving healthcare from private providers and a higher self-assessed health status. Despite these healthy habits, they also had higher rates of cardiovascular comorbidities (excluding hypertension) and a higher frailty index.

### Factors influencing vaccination uptake

Table 2 presents the multivariate logistic regression analysis results categorized according to cardiovascular comorbidities. Receiving an influenza vaccine within the 12 months preceding data collection was significantly associated with several factors. A higher likelihood of vaccination was observed among older individuals who were married/living with a partner, relied on private healthcare services, and had a good to excellent self-perceived health status. Conversely, factors such as black, higher alcohol intake, smoking status, and frequent engagement in vigorous physical activities were associated with a lower likelihood of vaccination. Although the subgroup analysis by comorbid cardiovascular conditions showed mixed results, age remained significantly associated with a higher likelihood of vaccination. Beyond age, several factors were significantly associated with vaccination in each subgroup. Notably, within the hypertension subgroup, relying on a private healthcare provider and having a good or excellent self-perceived health status were positively associated with higher vaccination odds. In contrast, c, and engaging in vigorous activities more than once a week were significantly associated with lower odds of being vaccinated. These associations were not observed among the chronic stable angina and myocardial infarction subgroups, whereas only a negative association with current smoking was observed in the heart failure subgroup.

### Reasons for vaccine hesitancy

Unvaccinated older adults expressed several common reasons for not receiving influenza vaccine. Personal perceptions of the vaccine accounted for 37.2% of the reasons, while contextual influences such as geographic barriers accounted for 17.9%. Reasons directly related to the vaccine or vaccination accounted for 21.5% of the cases. ( Central Illustration ) These included apprehension about adverse reactions (18.2%), belief in a low-risk infection (14.9%), vaccine unavailability (13.9%), and lack of confidence in its efficacy (12%). Figure 1 shows a bar graph illustrating the main reasons for not receiving influenza vaccine among those who were unvaccinated.

## Discussion

This nationwide study provides valuable insights into influenza vaccination rates in older Brazilian patients with cardiovascular comorbidities. Despite the Ministry of Health’s efforts to offer vaccines at no cost and a target vaccination coverage of 90%,^15^24% of this high-risk group remains unvaccinated, which poses substantial health risks. A higher likelihood of vaccination was noted among older individuals, those who were married or living with a partner, those using private healthcare, and those with good-to-excellent self-perceived health. Conversely, being black, higher alcohol intake, smoking, and frequent vigorous activity were linked to a lower likelihood of vaccination. Personal views on vaccines were the main factor influencing vaccination decisions, followed by contextual factors. Common reasons for not vaccinating included fear of adverse reactions perceived low risk of infection, vaccine unavailability, and doubts about efficacy. Beyond age, certain factors significantly influenced vaccination within the subgroups. In the hypertension subgroup, using private healthcare and positive self-health perception increased the odds of vaccination. Conversely, being black, consuming alcohol monthly or more, current smoking, and frequent vigorous activity were linked to lower vaccination odds. These patterns were not observed in the chronic stable angina and myocardial infarction groups; only current smoking negatively affected vaccination in the heart failure subgroup.

Our analysis revealed that vaccinated individuals were often older females utilizing private healthcare services, who also reported a positive self-assessment of their health. This pattern underscores the critical influence of healthcare access and individual health perceptions on vaccination behaviors.^21^These findings are consistent with existing literature highlighting how adults, particularly those engaged in the private healthcare system, are more likely to perceive the benefits of vaccination and act accordingly.^22^Older adults may recognize an increased risk of influenza complications and, thus, prioritize vaccination. By contrast, private healthcare access may provide more regular interactions with healthcare professionals who can advocate for vaccination.^22 , 23^Additionally, positive self-perceived health could correlate with proactive health behaviors and encourage vaccine uptake. Similar data were observed for individuals aged ≥ 60 years (n = 1,224) who participated in the 2015 SABE Study (Health, Well-Being, and Aging).^24^

Conversely, we found lower vaccination rates in demographics that included black individuals, those with higher alcohol consumption, smokers, and those engaged in vigorous physical activity. Lower vaccination rates among Black individuals may reflect social disparities and increased vulnerability, underscoring the need for targeted support to effectively address their unique needs.^22^This highlights the importance of culturally sensitive and inclusive public health initiatives that can effectively reach and support this demographic and ensure equitable access to healthcare resources and vaccination services. Similarly, the relationship between high alcohol consumption, smoking, and vaccination hesitancy suggests that targeted interventions are necessary to promote vaccination in these groups.^25 , 26^Interestingly, those engaging in vigorous physical activity might believe that they are less susceptible to illness due to perceived robust health, which necessitates greater emphasis on educating these populations about the importance of vaccination despite their healthy lifestyles.^27^

For individuals with hypertension, access to private healthcare and positive self-perception of health were correlated with a higher likelihood of receiving the influenza vaccine. This suggests that perceived health status and accessibility to healthcare resources are critical for promoting vaccination within this group. Conversely, lower vaccination rates were associated with being black, frequent alcohol consumption, smoking, and engaging in vigorous physical activity. These findings may indicate underlying socioeconomic and cultural influences, including possible barriers to healthcare access or differences in health beliefs that deter vaccination. Interestingly, these associations were not observed in the chronic stable angina and myocardial infarction subgroups, suggesting that the interplay of these factors may vary across different cardiovascular conditions. In the heart failure subgroup, only current smoking showed a negative association, potentially highlighting smoking as a key determinant of health behavior in this group.

Our study’s findings intersect with global health policy: In 2003, the World Health Assembly (resolution WHA 56.19) recommended boosting influenza vaccination coverage for high-risk individuals and aiming for ≥ 75% coverage among older individuals and those with chronic illnesses by 2010.^28^In Brazil, the Ministério da Saúde provides an influenza vaccine free of charge to older adults, with a target coverage rate of 90% by 2024.^14^According to our findings, Brazil successfully met the target of immunizing 75% of older adults with cardiovascular comorbidities. Similar data were observed in individuals aged ≥ 60 years (n = 23,815) who participated in the 2013 National Health Survey ( *Pesquisa Nacional de Saúde* (PNS))^29^and the 2015 SABE Study.^24^However, more information is needed to determine whether this was achieved in 2010 or whether our findings reflect a recent accomplishment. Notably, the influenza vaccine uptake surpassed the 75% threshold for all specific cardiovascular comorbidities studied, which was higher than the 50 to 60% uptake among high-risk older adults in other countries.^12 , 30 - 32^Despite these efforts, a 90% target has not been consistently achieved, indicating areas of improvement. These insights suggest that while Brazil has made significant progress in vaccination coverage for high-risk individuals, there remains the potential for further enhancement to meet national goals.

Furthermore, achieving vaccination targets relies on encouraging the uptake and understanding of the reasons driving vaccine hesitancy among unvaccinated individuals. Vaccine hesitancy remains a complex issue influenced by various underlying factors related to confidence and perceived risks.^33^An early study on polio vaccine hesitancy by Rosenstock et al.^34^identified factors that continue to be relevant. These factors include disease likelihood perception, disease severity judgment, vaccination efficacy assessment, and concerns and influences that affect decision-making. Our results showed similar traits among older adults with cardiovascular comorbidities: apprehension about adverse reactions, low self-risk perception of infection, and lack of confidence in vaccine efficacy highlighting inadequate communication regarding influenza and vaccination.

Moreover, a significant finding was that 13.9% of participants cited vaccine unavailability as a barrier, highlighting the logistical challenges in achieving optimal vaccination coverage. Addressing these barriers requires strategic planning, efficient distribution, and enhanced coordination between healthcare providers and government agencies to ensure consistent availability, especially during high-demand periods.^35 - 37^

Several studies have shown that healthcare providers are crucial for addressing and reducing vaccine hesitancy through clear and effective communication.^38 - 40^Healthcare providers who are well-informed and trained about vaccines are more likely to recommend vaccination.^23 , 40^However, additional support may be needed to handle challenging conversations with vaccine-reluctant patients or families, where societal endorsement and colleague support become essential.^23^The central illustration briefly summarizes how healthcare providers can address vaccine hesitancy.

To address these issues, it is crucial to investigate how older adults, especially those likely to remain unvaccinated, receive information regarding influenza and vaccination. Our study provided some insights into the unvaccinated population but highlighted the need for more detailed information on how older adults and their families are informed. Future studies should investigate influenza vaccine uptake in specific subgroups of older individuals and those with chronic illnesses.

Despite the insights provided, our study has several limitations. The cross-sectional design restricts our ability to establish causal relationships between vaccination status and the variables explored. Recall bias may affect the accuracy of self-reported vaccination data, potentially affecting reported rates. Additionally, reliance on self-reported cardiovascular disease data may introduce reporting bias. The exclusion criteria, focusing on Brazilians with cardiovascular conditions and missing data, could limit the generalizability of our findings to a broader population. Nevertheless, using a nationwide sample enhances the reliability and applicability of our results, thereby informing public health policies and interventions. Additionally, it is important to consider that the data collection from 2019 to 2021 coincided with the COVID-19 pandemic, which likely influenced the findings. The pandemic affected healthcare access, health-seeking behaviors, and vaccination priorities, impacting influenza vaccine uptake.^41 , 42^

## Conclusions

In conclusion, our study revealed that a significant proportion of older adults with cardiovascular comorbidities in Brazil remained unvaccinated against influenza. This underscores the necessity to increase vaccination efforts in this vulnerable population. Our findings suggest that tailored strategies addressing personal beliefs, access challenges, and healthcare provider engagement are essential for improving influenza vaccination. By closely aligning healthcare provider initiatives with individuals’ specific demographics and health characteristics, it is possible to mitigate the prevalent barriers to vaccination observed in this study.

**Table 1:** – Sociodemographic, Clinical, and Vaccination Characteristics of the Study Sample

Variable	Level	Overall	Unvaccinated	Vaccinated	p
		N = 5,296	N = 1,241	N = 4,055	
**Age, median [IQR]**	Years	67 [60, 75]	61 [56, 71]	68 [62, 76]	< 0.001
**Sex, %**	Female	3,059 (57.8)	686 (55.3)	2,373 (58.5)	0.047
	Male	2,237 (42.2)	555 (44.7)	1,682 (41.5)	
**Race, %**	White	2,361 (44.6)	502 (40.5)	1,859 (45.8)	0.001
	Black	619 (11.7)	174 (14.0)	445 (11.0)	
	Brown	2,270 (42.9)	558 (45.0)	1,712 (42.2)	
	Yellow/Indigenous	28 (0.5)	3 (0.2)	25 (0.6)	
	Unanswered	18 (0.3)	4 (0.3)	14 (0.3)	
**Marital status, %**	Not married	2,506 (47.3)	572 (46.1)	1,934 (47.7)	0.339
	Married/Living with a partner	2,790 (52.7)	669 (53.9)	2,121 (52.3)	
**Area of residence, %**	Rural	855 (16.1)	195 (15.7)	660 (16.3)	0.669
	Urban	4,441 (83.9)	1,046 (84.3)	3,395 (83.7)	
**Alcohol intake, %**	Never	4,318 (81.7)	969 (78.2)	3,349 (82.8)	< 0.001
	Less than once a month	407 (7.7)	93 (7.5)	314 (7.8)	
	Once a month or more	559 (10.6)	177 (14.3)	382 (9.4)	
**Smoking status, %**	Never smoked	3,518 (66.4)	768 (61.9)	2,750 (67.8)	< 0.001
	Current smoker	564 (10.6)	188 (15.1)	376 (9.3)	
	Former smoker	1,199 (22.6)	282 (22.7)	917 (22.6)	
	Unanswered	15 (0.3)	3 (0.2)	12 (0.3)	
**Engage in physical activity more than once a week, %**	Low intensity	2,284 (43.1)	529 (42.6)	1,755 (43.3)	< 0.001
	Moderate intensity	991 (18.7)	244 (19.7)	747 (18.4)	
	Vigorous intensity	459 (8.7)	144 (11.6)	315 (7.8)	
**Frequency of participation in vigorous physical activities such as running, swimming, cycling, doing aerobics or playing tennis, %**	More than once a week	459 (8.7)	144 (11.6)	315 (7.8)	< 0.001
	Once a week	137 (2.6)	44 (3.5)	93 (2.3)	
	1 to 3 times a month	68 (1.3)	14 (1.1)	54 (1.3)	
	Rarely or never	4,524 (85.4)	1,008 (81.2)	3,516 (86.7)	
	Unanswered	108 (2.0)	31 (2.5)	77 (1.9)	
**Healthcare provider, %**	Public	4,262 (80.5)	1,052 (84.8)	3,210 (79.2)	< 0.001
	Private	1,029 (19.4)	189 (15.2)	840 (20.7)	
	Unanswered	5 (0.1)	0 (0.0)	5 (0.1)	
**Self-assessed health status, %**	Very Poor	304 (5.7)	76 (6.1)	228 (5.6)	0.036
	Poor	816 (15.4)	202 (16.3)	614 (15.1)	
	Fair	2,225 (42.0)	530 (42.7)	1,695 (41.8)	
	Good	1,645 (31.1)	350 (28.2)	1,295 (31.9)	
	Very good	222 (4.2)	53 (4.3)	169 (4.2)	
	Excellent	83 (1.6)	30 (2.4)	53 (1.3)	
	Unanswered	1 (0.0)	0 (0.0)	1 (0.0)	
**Cardiovascular Comorbid Conditions, %**	Hypertension	5,103 (96.4)	1,190 (95.9)	3,913 (96.5)	0.444
	Chronic stable angina	188 (3.5)	39 (3.1)	149 (3.7)	0.044
	Myocardial infarction	420 (7.9)	91 (7.3)	329 (8.1)	0.030
	Heart failure	425 (8.0)	95 (7.7)	330 (8.1)	0.416
**Frailty assessment**	Frailty Index, median [ IQR]	0.20 [0.12, 0.32]	0.20 [0.12, 0.30]	0.21 [0.13, 0.32]	0.025
	Frailty level, %				
	Non-frail	925 (17.5)	238 (19.2)	687 (16.9)	0.136
	Pre-frail	1,737 (32.8)	410 (33.0)	1,327 (32.7)	
	Frail	2,634 (49.7)	593 (47.8)	2,041 (50.3)	

**Table 2:** – Multivariate Logistic Regression Results Stratified by Specific Subgroups of Cardiovascular Comorbidities.

Variable	Overall (N = 5,112)*	Cardiovascular Comorbid Conditions*			
		Hypertension (N = 4,927)	Chronic stable angina^**1**^(N = 179)	Myocardial infarction^**2**^(N = 389)	Heart failure^**3**^(N = 409)
**Age, years**	1.05 (1.05 - 1.06)	1.06 (1.05 - 1.06)	1.06 (1.01 - 1.11)	1.01 (0.99 - 1.04)	1.04 (1.01 - 1.07)
**Sex**					
Female	Reference	Reference	Reference	Reference	Reference
Male	0.93 (0.80 - 1.08)	0.92 (0.80 - 1.07)	1.09 (0.45 – 2.66)	0.72 (0.41 - 1.27)	1.46 (0.83 - 2.56)
**Race**					
White	Reference	Reference	Reference	Reference	Reference
Black	0.76 (0.61 - 0.93)	0.74 (0.60 - 0.92)	1.19 (0.31 - 4.57)	1.07 (0.49 - 2.35)	0.85 (0.38 - 1.92)
Brown	0.92 (0.80 - 1.07)	0.91 (0.78 - 1.06)	1.58 (0.66 - 3.77)	0.89 (0.51 - 1.54)	1.07 (0.63 - 1.84)
Yellow/Indigenous	2.53 (0.75 - 8.51)	2.24 (0.66 - 7.60)	-	-	-
**Marital status**					
Not married	Reference	Reference	Reference	Reference	Reference
Married/Living with a partner	1.17 (1.01 - 1.35)	1.16 (0.99 - 1.34)	1.34 (0.55 - 3.24)	1.44 (0.82 - 2.53)	1.38 (0.79 - 2.38)
**Area of residence**					
Rural	Reference	Reference	Reference	Reference	Reference
Urban	0.93 (0.77 - 1.12)	0.94 (0.77 - 1.14)	3.45 (0.96 - 12.42)	0.36 (0.12 - 1.09)	0.90 (0.41 - 1.96)
**Alcohol intake**					
Never	Reference	Reference	Reference	Reference	Reference
Less than once a month	1.15 (0.89 - 1.49)	1.13 (0.87 - 1.46)	0.23 (0.04 - 1.20)	1.62 (0.48 - 5.41)	0.62 (0.23 - 1.68)
Once a month or more	0.78 (0.63 - 0.97)	0.79 (0.64 - 0.99)	0.57 (0.15 – 2.20)	0.78 (0.32 - 1.92)	0.77 (0.31 - 1.93)
**Smoking status**					
Never smoked	Reference	Reference	Reference	Reference	Reference
Current smoker	0.68 (0.55 - 0.83)	0.70 (0.57 - 0.87)	0.46 (0.13 - 1.58)	0.69 (0.34 - 1.40)	0.42 (0.20 - 0.88)
Former smoker	0.91 (0.77 - 1.08)	0.90 (0.76 - 1.07)	1.00 (0.40 - 2.50)	0.94 (0.53 - 1.67)	1.34 (0.76 - 2.39)
**Frequency of participation in vigorous physical activities**					
Rarely or never	Reference	Reference	Reference	Reference	Reference
1 to 3 times a month	1.09 (0.59 - 2.02)	1.17 (0.62 - 2.20)	-	-	1.23 (0.13 - 11.40)
Once a week	0.72 (0.49 - 1.06)	0.71 (0.48 - 1.04)	-	0.94 (0.09 - 9.93)	1.39 (0.16 - 12.20)
More than once a week	0.72 (0.58 - 0.90)	0.74 (0.59 - 0.93)	0.82 (0.23 – 2.90)	0.34 (0.13 - 0.92)	0.68 (0.30 - 1.54)
**Healthcare provider**					
Public	Reference	Reference	Reference	Reference	Reference
Private	1.29 (1.08 - 1.56)	1.30 (1.08 - 1.58)	0.89 (0.34 - 2.31)	1.67 (0.85 - 3.30)	1.42 (0.75 - 2.71)
**Self-assessed health status**					
Very Poor/Poor	Reference	Reference	Reference	Reference	Reference
Fair	1.14 (0.94 - 1.39)	1.17 (0.95 - 1.42)	0.68 (0.26 - 1.77)	0.97 (0.53 - 1.77)	1.08 (0.59 - 1.96)
Good/Excellent	1.28 (1.02 - 1.61)	1.33 (1.06 - 1.69)	0.48 (0.14 - 1.65)	1.52 (0.68 - 3.39)	1.10 (0.48 - 2.51)
Frailty Index score	0.68 (0.37 - 1.25)	0.74 (0.40 - 1.37)	0.33 (0.01 - 9.15)	0.61 (0.10 - 3.92)	0.53 (0.08 - 3.47)

*Estimates are presented as Odds Ratios (95% Confidence Intervals). 1 To prevent biased estimates resulting from a small number of observations, we excluded 2 cases (“Yellow/Indigenous” race and “1 to 3 times a month” frequency of participation in vigorous physical activities) from the “Chronic stable angina” subgroup. 2 To prevent biased estimates resulting from a small number of observations, we excluded 2 cases (“Yellow/Indigenous” race and “1 to 3 times a month” frequency of participation in vigorous physical activities) from the “ Myocardial infarction” subgroup. 3 To prevent biased estimates resulting from a small number of observations, we excluded 2 cases (“Yellow/Indigenous” race) from the “ Myocardial infarction” subgroup.

## *Supplemental Materials

For additional information, please click here.
